# Powder milk: a user-friendly and safe product for heated-milk food challenge?

**DOI:** 10.1186/s13223-015-0103-z

**Published:** 2015-12-23

**Authors:** Sabrine Cherkaoui, Philippe Bégin, Louis Paradis, Jean Paradis, Anne Des Roches

**Affiliations:** Divison of Clinical Immunology and Allergy, Department of Medicine, University of Montreal, Montreal, QC Canada; Division of Pediatric Allergy and Immunology, Department of Pediatrics, Sainte-Justine University Hospital Center, University of Montreal, 3175, Chemin de la Côte Sainte-Catherine, Montreal, QC H3T 1C5 Canada

**Keywords:** Heated milk, Baked milk, Milk allergy, Tolerance, Introduction, Powder milk, Challenge

## Abstract

**Background:**

Previous studies have reported that up to 75 % of milk allergic subjects tolerate heated milk products. However, the food used for heated milk challenge is often prepared in a non-standardized manner by the parents at home, which may prove inconvenient and even sometimes raise concerns with regards to test validity. Instant skim milk powder is made by a food process that involves heating skim milk to up to 250 °C (390 °F) for up to 30 min which ought to be sufficient to denature thermo-labile proteins.

**Objective:**

To appraise the use of instant skim milk for the purpose of heated milk food challenge.

**Methods:**

We reviewed all oral food challenges to instant skim milk powder performed at Sainte-Justine University Hospital Center in Montreal, Canada between November 2008 and January 2013 (cumulative dose of 4 g proteins).

**Results:**

During the study period, 39 children underwent an open food challenge to instant skim milk powder. Thirty patients (76.9 %) passed the challenge without clinical reaction, of which 26 successfully introduced heated milk products at home. The remaining four children reported intermittent mild reactions to specific forms of heated milk goods while they tolerated others. Subjects’ clinical and paraclinical characteristics were comparable to previous cohorts evaluating baked milk challenge, which reported similar rates of heated milk positive challenges, ranging from 17 to 28 %.

**Conclusion:**

Challenge with instant skim milk powder could be a safe, convenient and easily standardizable alternative to home baked food for heated milk challenge. Further controlled studies are needed before this can be implemented to practice.

## Background

Cow’s milk protein allergy is the most common food allergy among children, affecting 1–3 % [1, 2] of young children and causing up to 13 % of fatal food induced anaphylaxis [3]. It is caused by IgE antibodies directed against a wide variety of different sequential or conformational epitopes on different milk proteins [4]. Since food heating can affect protein conformation and modify conformational epitopes, it can drastically reduce reactivity in those subjects with antibodies directed mainly against such conformational epitopes [5]. This explains why a majority of milk allergic children will tolerate heated milk in baked products [1, 2, 6, 7].

Identifying children that can tolerate heated milk products is extremely important given current management of food allergy relies on strict avoidance of food allergens. Because it is present in so many processed foods, cow’s milk protein allergy is associated with a major burden for patients and families [8]. Its negative impact on quality of life [9] and the burden of cow’s milk diet exclusion [10] have been well documented. Identification of heated milk tolerance can lead to a relaxation of the diet, making it easier to follow. In addition, the regular introduction of heated milk into the diet could accelerate the development of fresh milk tolerance [1]. Evaluation of tolerance to heated milk is thus an extremely important part of the management of milk allergic children [11].

Heated milk challenge is usually performed with muffins or cupcakes [1, 6, 12] made with nonfat milk powder baked at 350 °F for 30 min, containing up to 2.6 g of milk protein. Other reported alternatives have included waffles and well-cooked cheese on a pizza baked at 425 °F for at least 13 min [1] (made with 4.6 g of milk protein), rice pudding baked at 325 °F for 90 min (containing 7.7 g of milk protein) [13] and a variation of the traditional approach with muffins baked at 180 °C (360 °F) for 20 min (containing 0.5 g of milk protein) [14].

In our center, we have used a different heated milk challenge technique consisting of a glass of reconstituted milk from instant skim milk powder. We postulate that the performance of our challenge technique, which is user-friendly as it is easy and rapid to prepare and allows verifying the exact intake of milk protein, is comparable to other techniques previously published.

## Methods

Charts of all patients who underwent oral food challenges to instant skim milk powder at Sainte-Justine University Hospital Center in Montreal from November 2008 through January 2013 were reviewed. To be included in the analysis, the patients needed a prior history of allergic reaction to milk and detectable milk proteins IgE as determined by a positive skin prick test (SPT) (3 mm greater than control) and/or serum milk-specific IgE levels ≥0.35 kU/L (ImmunoCAP, Phadia, Upsala, Sweden) or >0.1 kU/L (Immulite, Siemens Healthcare Diagnostics, Tarrytown, NY). No upper limit for specific IgE levels and SPT values was set for study exclusion.

Patients who had already tolerated heated milk products introduced at home were excluded. The study was approved by our institutional ethics committee.

SPTs were performed according to previously published methods [1] using commercial extracts (Omega Laboratories LTD, Montreal, Canada) for milk, α-lactalbumin and casein. We also performed SPTs with fresh cow’s milk and instant skim milk powder described below. Control tests for SPTs were performed with histamine (positive control) and a normal saline (negative control). Wheal diameters were measured after 15 min. A positive SPT was defined as a wheal larger than 3 mm than the negative control.

After obtaining consent from parents, open food challenges to heated milk were performed using a glass of milk reconstituted from instant skim milk powder (Compliment Instant Skim Milk Powder, Sobeys, Mississauga, Ontario © or Carnation Instant Skim Milk Powder, Smuckers Foods of Canada Corp, Markham, Ontario ©). The challenge consisted in a step-wise ingestion of progressive amounts (0.5, 5, 30 and 85 mL) up to a total of 120 mL (approximately four ounces) of powder milk mixed with water, which is equivalent to 4 g of milk protein. In children who did not want to drink the powder milk preparation, it was mixed with chocolate. Written consent was provided before the procedure.

Children were observed in the allergy clinic during the challenge and at least 1 h after the last dose of powder milk. The challenge was stopped if symptoms occurred and the child was re-examined by the physician and treated accordingly. Anaphylaxis was defined according to World Allergy Organization criteria [15]. Our institution’s protocol was to introduce heated milk products at home if the patient tolerated the challenge.

Parents were advised to introduce regularly in the diet baked cow’s milk goods that were well cooked in the oven such as cakes, muffins and brownies. Patients were also allowed to consume recipes with powder milk (milkshakes, sauces, cakes, etc.) but fresh cow’s milk has to be strictly avoided in the diet.

We conducted a literature review on Medline using the following keywords: baked milk challenge, powder milk challenge, heated milk challenge, baked milk tolerance, and heated milk tolerance. The Wilcoxon rank-sum test was used to compare continuous baseline characteristics between heated milk tolerant and reactive patients. The two-sample χ^2^ test was used to compare categorical data.

## Results

Forty-four children underwent a challenge to instant skim milk powder during the study period. Two patients were excluded because they did not complete the challenge because of food aversion even though no objective allergic reaction occurred. Two patients were further excluded because SPT to extracts and fresh cow’s milk were negative. Lastly, one patient was excluded from the diet tolerance analysis because his parents chose not to introduce heated milk products at home despite recommendation because of fear of having an adverse reaction. As a result, a total of 39 milk allergic patients (median age 9 years, range 2–17) having undergone instant skim milk powder were included into the analysis. Of these, 30 (76.9 %) passed the initial OFC. Characteristics of heated milk tolerant and heated milk allergic are compared in Table 1.

**Table 1:** Study participants characteristics

Outcomes of the oral challenge	Heated milk reactive	Heated milk tolerant	p value
No of patients (%)	9 (23.0)	30 (76.9)	
Reaction threshold (mg protein), median [range]	1.25 [0.0036–4]	NA	
Age, median (years) [range]	9 [5–15]	9 [2–17]	.75
Sex, no (%)			
Male	6 (66)	17 (57)	.59
Median specific IgE levels (kU_A_/L) [range]			
Cow’s milk	3.41 [0.89 to >100]	0.74 [<0.10–3.53]	.004
Casein	1.20 [0.51–80.8]	0.29 [<0.10–3.65]	.006
β-Lactoglobuline	1.12 [0.55–1.59]	0.51 [0.18–1.44]	.16
α-Lactalbumine	3.83 [0.55–44.6]	0.66 [<0.10–1.97]	.01
Total IgE	611 [58–1882]	206 [32–2470]	.75
Median SPT wheal (mm) [range]			
Fresh cow’s milk	7.5 [4–16]	7.5 [4–13]	.42
Powder milk	11.5 [8–13.5]	7.25 [0–13]^†^	.55
Cow’s milk extract	5 [3–10.5]	4 [0–12.5]^†^	.32
Casein	6 [3–11]	4 [3–9.5]	.96
α-Lactalbumin	5.5 [0–8.5]	3.5 [0–14]^†^	.79
Index reaction to milk involving			
Skin	6 (66 %)	14 (47 %)	.29
Airway	1 (11 %)	7 (23 %)	.43
GI tract	8 (89 %)	20 (66 %)	.04
Hypotension	1 (11 %)	0	.07
Systemic anaphylaxis	2 (22 %)	9 (30 %)	.65
Other atopic conditions, no (%)			
Asthma	3 (33)	16 (53)	.29
ARC	4 (44)	10 (33)	.70
Atopic dermatitis	6 (67)	22 (73)	.70
Other food allergies	8 (89)	28 (93)	.66

*ARC* allergic rhinoconjunctivitis, *SPT* Skin Prick Test

^†^Three negative SPTs for powder milk, milk extract and lactalbumin were observed in three separate patients

Briefly, there were 6 (66.7 %) males in the heated milk reactive group compared to 17 (56.7 %) in the heated milk tolerant group. The prevalence of collateral atopic manifestations (asthma, atopic dermatitis, allergic rhinoconjunctivitis and other food allergies) did not differ between the two groups. When comparing initial cow’s milk reaction, children reactive to heated milk were more likely to report a history of gastrointestinal tract symptoms to milk than heated-milk tolerant (p < 0.04). There was no significant difference noted for other systems. Only one child had a history of hypotension to cow’s milk, which was found to be heated milk reactive (p = 0.07).

Interestingly, no difference was found in SPT results (cow’s milk extract, casein extract, α-lactalbumin extract, powder milk, fresh cow’s milk) between those who passed and those who failed instant powder milk challenge. However, cow’s milk specific IgE levels were significantly higher in patients who failed baked milk challenge (p < 0.004). Casein and α-lactalbumin specific IgE levels were also significantly higher in heated milk reactive children (p < 0.006 and p < 0.01, respectively).

Most reactions to instant skim milk powder challenge were found to be mild and responded well to anti-histaminic drugs. The median reaction threshold was 1.25 g of milk protein, ranging from 0.0036 to 4 g. More than half of heated milk reactive children (55.6 %) had oral and/or oropharyngeal pruritus. Four (44.4 %) subjects presented gastrointestinal symptoms. Only one patient (patient 7) had immediate generalized hives and recurrent vomiting that required the injection of intramuscular epinephrine. This patient recovered shortly after a single dose of epinephrine and was discharged home few hours later. No patient had cardiovascular or respiratory symptoms during the challenge. Heated milk reactions are detailed in Table 2.

**Table 2:** Failed heated milk challenge

Patient	SPT wheal (mm)			Serum antigen specific IgE (kU_A_/L)				Prior reaction	Symptoms during OFC	Total amount (g protein)	Treatment
	Casein	Fresh milk	Powder milk	α-Lactalbumin	β-Lactoglobulin	Casein	Cow’s milk				
1	NA	5	8	1.33*	NA	0.828*	1.94*	Hives, vomiting	Facial flushing, conjunctival erythema, abdominal pain	4	Antihistaminic
2	3.5	5.5	13.5	5.04*	1.17*	1.32*	6.8*	Atopic dermatitis, vomiting	Oral pruritus	0.8	Antihistaminic
3	3	6		1.82**	2.42**	2.57**	5.33**	NA	Oral pruritus, hives, sneezing	1.84	Antihistaminic
4	NA	4	7.5	0.545*	0.555*	0.505*	0.899*	Vomiting oral pruritus	Pharyngeal pruritus persistent post challenge	2	None
5	11	16	15.5	5.87*	1.59*	2.55*	4.51*	Generalized hives, vomiting, dyspnea, hypotension	Uvula angioedema, throat pain	0.0296	Antihistaminic
6	4.5	9	8	2.62*	1.06*	1.08*	2.3*	Generalized hives, face angioedema, diarrhea	oropharyngeal & ear pruritus	0.0036	Antihistaminic
7	6.5	9	13	44.6*	NA	80.8*	>100*	Generalized hives, vomiting	Generalized hives & pruritus, vomiting	1	Epinephrine + antihistaminic
8	6	10	10	2.82**	4**	1.99**	3.86**	Hives, facial angioedema, vomiting	Vomiting, abdominal pain	1.5	Antihistaminic
9	6.5	7.5	13	NA	NA	NA	NA	Hives, diarrhea	Vomiting	1.3	Antihistaminic

*NA* not available

* Immulite IgE dosage technique

** Unicap IgE dosage technique

We conducted a phone follow-up among subjects that had passed instant powder milk challenge to evaluate whether baked milk products had been introduced and tolerated at home. We were able to contact 24 of 30 heated milk tolerant patients (80 %). The remaining 6 subjects who could not be reached by phone all had had a clinic follow-up visit after the challenge in which physician notes attested that they were eating various types of heated milk products without any allergic reaction.

The majority (86.7 %) of baked milk tolerant children was frequently eating various types of heated milk products such as brownies, cakes, muffins, and manufactured baked mozzarella cheese without any clinical reaction. Four patients reported intermittent and mild reactions to specific forms of heated milk products that responded well to antihistamines but tolerated other forms (Table 3). Curiously, we did not identify an association between the milk protein’s cooking degree and the symptoms reported by those four patients. For example, patient number 2* tolerated baked cheese but did not tolerate brownies that were cooked longer in the oven. Briefly, two children reported mild gastrointestinal symptoms: one had abdominal pain and regurgitation that resolved spontaneously and one had vomiting that responded to oral antihistamine treatment. The remaining two patients had symptoms that could not be objectified; one reported throat pain that resolved with antihistamines and the other expressed anxiety and experienced tongue pruritus that resolved completely without any medication. There was no systemic anaphylaxis with baked milk introduction and no subject required epinephrine injection at home.

**Table 3:** Children partially tolerant to heated milk after home reintroduction

Patient	Age (years)	Type of heated milk tolerated	Type of heated milk not tolerated	Symptoms during reintroduction of heated milk	Treatment
1*	12	Muffins, cakes, brownies, ice cream	Baked cheese (all sorts)	Abdominal pain, regurgitation	None
2*	9	Brownies, chips, crackers, cakes	Mozzarella cheese, pudding, powder milk contained in sauce and milkshake	Throat pain	Antihistaminic
3*	17	Baked cheese (pizza)	Powder milk ingredients (brownies etc.)	Tongue pruritus anxiety	None
4*	15	Bread containing powder milk	Powder milk ingredients (brownies etc.)	Vomiting	Antihistaminic

## Discussion

Recent advances in food allergies have shown the importance of evaluating the tolerance to heat-denatured milk proteins as part of the management of cow’s milk protein allergic children in order to relax the avoidance diet but also to change the natural history of cow’s milk protein allergy [16]. To our knowledge, this is the first study to report the use of instant skim milk powder as a tool for heated milk challenge to this effect. Our experience has shown this approach to be safe, efficient and user-friendly.

In heated milk reactive patients, challenge reactions were mostly mild, except for one child who required epinephrine administration. In patients who tolerated the heated milk challenge, there was no serious reaction upon reintroduced at home. The dose of milk protein reached with this protocol (4 g) thus appears to allow a safe reintroduction of heated milk products at home. Four subjects (13 %) reported partial tolerance of baked milk products at home. The basis for these reactions remains unclear as the patients would report reactions to foods with lower milk amounts having undergone longer heating times. It is possible that changes in baseline reaction threshold (from infection, exercise, pollen season, menses) could explain reaction variability, independent of heated milk dose.

After performing a Medline search of the keywords mentioned in the “Methods”, we found 68 articles (date: December 2014). Of these, six studies presented detailed original results of heated milk oral food challenges and are described in Table 4.

**Table 4:** Summary of studies on heated milk challenge

Study	Population	Median age (years)	Design	Type of HM OFC	HM tolerant	HM reactive	Milk SPT, median (mm) (range) HM tolerant	Milk SPT, median (mm), median (range) HM reactive	Milk sIgE (kU_A_/L), median (range), HM tolerant	Milk sIgE (kU_A_/L), median (range), HM reactive	Casein sIgE (kU_A_/L) median (range) HM tolerant	Casein sIgE (kU_A_/L)median (range) HM reactive	Main findings
Nowak et al. [4]	Eligible subjects aged 0.5–21 years, with positive SPT responses or detectable serum milk-specific IgE, and had a history of an allergic reaction to milk within 6 months before study entry or milk-specific IgE levels or SPT responses greater than 95 % of predicted value for clinical reactivity N = 100	7.5 (2.1–17.3)	Prospective	Muffin (baked at 350 F for 30 min in an oven) and waffle containing 1.3 g milk protein (cooked in a waffle maker at 500 F for 3 min) Total 2.6 g of CM protein	77 (77 %)	23 (23 %)	7 (2.5–19)	9.5 (5–24)	2.43 (0–79.1)	11.6 (0.69–101)	1.4 (0–101)	14.15 (0.71–101)	Among 100 children who undertook HM challenges: 68 tolerated extensively HM only, 23 reacted to HM, and 9 tolerated both heated and unheated milk. HM reactive children had significantly larger SPT wheals and higher milk-specific and casein-specific IgE levels than other groups
Kim et al. [1]	Eligible subjects same as [4] N = 89 Comparison group matched to active subjects (N = 60)	6.6 (2.1–17.3)	Prospective	Each muffin contained 1.3 g of milk protein (baked at 350 F for 30 min). And cheese pizza containing 4.6 g of milk protein (baked at 425 F for 13 min or longer)	65 (74 %)	23 (26.1 %)	NA	NA	NA	NA	NA	NA	Among 65 children initially tolerant to HM, 39 (60 %) now tolerate unheated milk,. Among the HM reactive group (n = 23), 2 (9 %) tolerate unheated milk, 3 (13 %) tolerate HM and baked cheese, whereas the majority (78 %) avoid milk strictly. Children initially tolerant to HM were more likely to become unheated milk tolerant compared with HM reactive children (p < 0.001) and those who incorporated dietary baked milk were more likely than the comparison group to become unheated milk tolerant (p < 0.001)
Caubet et al. [17]	Two cohorts of milk allergic children N = 97 from [4] and a second cohort of N = 128. Eligibility criteria same as [4] and [1] Total N = 225	Second cohort HM tolerant: 7.5 (4.0–11.0) HM reactive 8.0 (4–10)	Prospective	Same as [4]	83 (64.8 %)	38 (29.7 %)	NA	NA	(0.2–42.3)	11.9 (0.8–50.5)	2.3 (0.2–30.5)	12.2 (0.5–67.0)	The two cohorts of milk allergic children demonstrated the levels of IgE to cow’s milk, casein and β-lactoglobulin were significantly higher in HM reactive patients compared with HM tolerant patients. Casein-specific IgE had the highest positive and negative predictive values compared with specific IgE to cow’s milk or b-lactoglobulin, and casein-specific and b-lactoglobulin specific IgE/IgG4 ratios were significantly higher in HM reactive children with compared with HM -tolerant children
Ford et al. [13]	Eligible subjects were between the ages of 4 and 10 years and had a positive SPT response to milk or detectable serum milk-specific IgE levels and a history of an allergic reaction to milk N = 132	7.6 (4.0–11.0)	Prospective	Muffin same as [4] pizza (4 g of milk protein baked at 425 F for at least 13 min), rice pudding (7.7 g of milk protein baked at 325 F for 90 min)	95 (72 %)	37 (28 %)	NA	NA	NA	12.4 (0.6–43.6)	NA	13.75 (0.36–49.9)	The majority of patients with milk allergy are able to tolerate some forms of HM in their diets. Casein- and milk-specific IgE level, milk-specific basophil reactivity, and milk SPT wheal diameter are all significantly greater among patients with milk allergy who react to HM than among those who tolerate it
Bartnikas et al. [12]	All patients had a history of prior allergic reactions to milk (either baked or unheated) documented in the medical record by an allergist and detectable milk protein sIgE, as determined by a positive SPT result or elevated serum sIgE level. N = 35	8.1 (3.1–18)	Retrospective	Each muffin or cup-cake of milk protein (baked at 350 F for 30 min) (total of 2.6 g of milk protein)	29 (83 %)	6 (17 %)	10 (0–20)	15 (7–20)	1.93 (<0.35–20.6)	2.39 (<0.35–31.0)	1.05 (<0.35–10.3)	1.07 (<0.35–31.5)	Most children allergic to cow’s milk tolerated baked milk. Milk protein SPT wheal may be more reliable than sIgE level in predicting outcomes of baked milk challenges. There is a possibility of late reactions to ongoing baked milk exposure at home
Mehr et al. [14]	Previous convincing clinical reaction to CM with SPT reaction or sIgE to CM N = 70	HM tolerant 4.5 (2.5–8) HM reactive 7.3 (4.9- 7.3)	Not mentioned	A standard recipe was used for the muffin, baked at 180 ℃ for 20 min (containing 0.5 g of cow’s milk protein)	51 (72.9 %)	19 (27.1 %)	8 (7.0–10)	8.5 (7.5–10.0)	NA	NA	NA	NA	51 (73 %) passed the OFC and successfully incorporated baked CM into their diet. 19 children (27 %) reacted to their challenge. Of reactors, 4 (21 %) developed anaphylaxis and required intramuscular adrenalin. Predictors of clinical reactivity to baked CM were asthma, asthma requiring preventer therapy, IgE mediated clinical reactions to >3 food groups, and those with a history of CM anaphylaxis
This study	To be included, the patients should have a prior history of allergic reaction to milk and detectable milk protein sIgE as determined by a positive skin prick test (SPT) and/or elevated serum milk-specific IgE. N = 39	9 (4.0–17.0)	Restrospective	A glass of instant skim milk powder that was equivalent to a total of 4 g of cow’s milk protein (approximately 120 mL)	30 (76.9 %)	9 (23.1 %)	7.5 (4–13)	7.5 (4–16)	0.86 (<0.35–9.8)	4.19 (0.89–>100)	0.38 (<0.35–3.65	1.65 (0.51–80.8)	30 (76.9 %) passed the powder milk challenge. Compared to those who were HM tolerant, HM reactive children had higher specific IgE levels to cow’s milk (p < .004), casein (p < .006) and α-lactalbumin (p < .01). In comparison to other studies on HM challenge, our study demonstrates comparable children characteristics. This study shows a similar rate of positive challenge to HM compared to previously published studies and demonstrates a new technique of HM challenge that is user-friendly and safe

Using this instant powder milk challenge approach, we obtained a rate of 23.1 % of positive challenge, which is comparable to other heated milk challenge techniques used in other studies with similar populations, which have been summarized in Table 4 [1, 6, 14, 17]. The Nowak-Wegrzyn group [6], which used muffins and waffles containing heated milk, reported an identical positive challenge rate of 23 %. Bartnikas and colleagues [12], which used heated milk challenges to muffins or cupcakes had a positive challenge rate of 17 %. In their report, Ford and colleagues [13] identified 28 % of heated milk reactive children using a series of heated milk challenges including muffin, pizza and rice pudding. Finally, a recent study by Mehr and colleagues [14] showed that 27 % children reacted to heated milk in the form of a baked muffin containing 0.5 g of milk protein.

Given the study’s retrospective design, we cannot exclude that some subjects that were found to be heated milk tolerant may have also been fresh milk tolerant, having already outgrown their milk allergy at time of challenge. To overcome this limit, a second prospective study would be warranted where powder milk tolerant children would undergo fresh cow’s milk challenges to prove that they are still milk allergic.

This said, the reported reaction rate is a reflection of real-life clinical approach in which heated milk is normally introduced at home before attempting fresh milk challenge. The concordance with previous reports further supports the idea that clinicians should expect heated milk challenge reaction rates to be between 17 and 28 % in this population.

It is possible that patients who reacted to powder milk could have been baked goods tolerant. A follow up study is warranted where all powder milk reactive children are challenged to baked milk goods within a week.

Despite heating powder milk may not be completely equivalent to baked foods. Another hypothesized mechanism that reduce the allergenicity in baked foods is the formation of disulfide bonds that may modify IgE binding and thus allergen presentation to the immune system. For example, beta-lactoglobulin protein when heated will bind to other food proteins of the matrix and this will reduce its allergenic potential [18]. For example for egg, it has been suggested that ovomucoid is polymerized by heating and forms complexes with gluten that leads to markedly insoluble ovomucoid [19].

Heated milk reactive children had higher median specific IgE levels to cow’s milk protein, casein, α-lactalbumin and β-lactoglobulin. This was also consistent with findings from previous studies [6, 17]. Specifically, Nowak and colleagues [6] have shown that patients with milk-specific IgE > 35 kU_A_/L had about 85 % chance of reacting during heated milk challenge and a decision point of 5 kU_A_/L demonstrated approximately 90 % rate of heated milk challenge tolerance. Another study [17] demonstrated that a casein-specific IgE cutoff of 0.94 kU_A_/L had a negative predictive value of 96 % but a low specificity of 32 % to determine a negative decision point.

Use of reconstituted instant powder milk for challenge offers practical advantages over baked food. The main method for drying milk in the dairy industry is spray drying, which involves heating to up to 200 °C (390 °F) for up to 30 min, thus meeting generally accepted criteria for “heated milk” [20–22]. For the parents, it eliminates the worry of properly baking the challenge product at home, which can be stressful, as evidenced by parents who prepare multiple cakes “to make sure they get it right”. This preparation requires fresh milk manipulation at home and could be associated with a potential contamination of the environment and accidental contact with this allergen. Preparation with instant milk powder is quick and easy. As powder milk is easily stored, challenges can be performed immediately when clinical criteria are met instead of having to schedule another appointment for parents to bake the cake. For the clinician, there is the advantage of knowing precisely the amount of milk that is being administered to the patient whilst it is much more approximate in baked goods. Parent’s errors at home can lead to low amounts of milk in challenge product which could in turn lead to false reassurance after passed challenge. Even when the parent’s calculations are correct, it often remains difficult to precisely ascertain the exact amount of milk that was ingested.

Beyond its practical aspects for heated milk challenge, this study raises the issue that instant powder milk may not be equivalent to fresh milk from an antigenic point of view. This notion could be relevant in other settings such as oral immunotherapy. While successful use of powder milk has been reported, to the best of our knowledge it has never been directly compared to fresh milk in that context [23, 24].

## Conclusion

We have shown that heated milk challenge using a glass of milk prepared with instant milk powder could be a safe, efficient and convenient method for patients, families and physicians to test for heated milk tolerance compared to the standard which could be considered as a tool in both research and clinical settings. Further controlled studies are needed before this can be implemented to practice.

## Abbreviations

OFC: oral food challenge
SPT: skin prick test
CM: cow’s milk
HM: heated milk
